# The expression of decision and learning variables in movement patterns related to decision actions

**DOI:** 10.1007/s00221-024-06805-y

**Published:** 2024-03-29

**Authors:** Ida Selbing, Joshua Skewes

**Affiliations:** 1 Division of Psychology, Karolinska Institutet, Nobels väg 9, Solna, Stockholm, Sweden; 2 Department for Linguistics, Cognitive Science, and Semiotics, Aarhus University, Aarhus, Denmark; 3 Interacting Minds Centre, Aarhus University, Aarhus, Denmark

**Keywords:** Decision-making, Action dynamics, Learning, Bayesian inference, Movement

## Abstract

Decisions are not necessarily easy to separate into a planning and an execution phase and the decision-making process can often be reflected in the movement associated with the decision. Here, we used formalized definitions of concepts relevant in decision-making and learning to explore if and how these concepts correlate with decision-related movement paths, both during and after a choice is made. To this end, we let 120 participants (46 males, mean age = 24.5 years) undergo a repeated probabilistic two-choice task with changing probabilities where we used mouse-tracking, a simple non-invasive technique, to study the movements related to decisions. The decisions of the participants were modelled using Bayesian inference which enabled the computation of variables related to decision-making and learning. Analyses of the movement during the decision showed effects of relevant decision variables, such as confidence, on aspects related to, for instance, timing and pausing, range of movement and deviation from the shortest distance. For the movements after a decision there were some effects of relevant learning variables, mainly related to timing and speed. We believe our findings can be of interest for researchers within several fields, spanning from social learning to experimental methods and human–machine/robot interaction.

**Supplementary Information:**

The online version contains supplementary material available at 10.1007/s00221-024-06805-y.

## The expression of decision and learning variables in movement patterns related to decision actions

If you reach for a fruit from a fruit basket, or decide which one of two cords to cut in order to disarm a bomb, will your movements somehow reflect your underlying decision process? Perhaps your movement will be affected by how confident your decision is, or whether or not you expect the consequences of your decision to be positive or negative.

Research focusing on how decisions unfold over time would argue that the planning and execution of a decision are not always possible to separate (Freeman 2018). Rather, the decision-making process is often reflected in movement patterns or spatiotemporal features of the decision action (Gallivan et al. 2018). A typical example is that movements often deviate towards the distractor target when there is more than one target object available, suggesting that objects initially compete for action selection (Welsh & Elliott 2004). This dynamic view of decision actions is further supported by studies on the neural mechanisms of decision-making (Cisek 2007; Klein-Flugge & Bestmann 2012).

If the decision, and possibly also learning, processes are expressed in the movement features of a decision action, this not only provides us with a method to study or measure the underlying processes, it can also have real-life consequences. Expression of aspects of the decision process might for instance be relevant in social interactions. For example, it has previously been demonstrated that communication of choice confidence is important during joint decision-making (Bahrami et al. 2010). During social interactions, it would also be valuable for an observer to be able to infer if the outcome of someone else’s decision is better or worse than s/-he predicted. From this point of view, it would be valuable for an observer if the prediction error (Den Ouden et al. 2012; Niv & Schoenbaum 2008), the difference between the expected and the actual outcome of an action, would be expressed in the decision action. Both choice confidence and prediction errors, as well as other aspects of decision-making and learning, can be formally defined using computational methods. Research investigating the link between decision processes and movement patterns have however typically not formalized the decision process. As an example, researchers demonstrating effects of ambivalence on deviations in movement trajectories during decision actions used a paradigm where ambivalence relied on participants’ classifications of objects as either positive or negative (Schneider & Schwarz 2017), rather than a formal definition.

One way to formalize decision-making is through reinforcement learning (RL) modeling, which models how an agent acts and learns to make decisions through trial and error (Sutton & Barto 1998). RL modeling is a standard approach to understand how humans and non-human animals make decisions from experience (Charpentier et al. 2021; Dayan & Daw 2008; Lee et al. 2004). One of the strengths of RL as a framework is the possibility to link RL algorithms with neural mechanisms (Niv 2011), often by comparing latent variables in the algorithm, such as prediction errors to for instance, neuroimaging data (Daw & Doya 2006), or pupil dilation data (Nassar et al. 2012; Stemerding et al. 2022).

A commonly used, simple and non-invasive way to measure movement patterns related to decision actions in humans is through mouse-tracking (Freeman 2018), a method where the movements of a mouse cursor are recorded during some computerized task. Mouse-tracking has mainly been used to study the process of judgments and categorical decision-making (Freeman et al. 2008), but occasionally also value-based decisions (Cheng & González-Vallejo 2018).

## Aim of the present study

The aim of the work presented here is to explore the relation between computationally inferred decision and learning variables and the spatiotemporal features of a decision action. These phenomena are inherently complex. In particular, the spatiotemporal features of even simple actions lend themselves to a broad range of modelling and measurement strategies. Rather than focus on a limited set of measurements, we have therefore elected to sample broadly, in an attempt to identify which specific spatiotemporal aspects of mouse movement may be reflected in choice contexts. As such, our research strategy is one of exploration rather than explicit hypothesising. To this end, we designed a simple computerized probabilistic decision-making task that would allow us to compute, trial-by-trial, decision and learning variables of interest. These variables could then be correlated with movement data, as measured by mouse-tracking, thus providing a new window into how learning and decision-making affects the execution, and not just the selection, of an action. To do this we constructed a computational RL model based on Bayesian inference, which allowed us to extract several latent key decision and learning variables, for instance choice confidence and prediction errors.

In order to deepen our understanding of what aspects of the decision and learning process are expressed in movements, we analyzed the correlation between movement patterns and several decision and learning variables, more specifically 1) the confidence with which a decision was made and how it changed as a result of learning, 2) the value of the decision context (mean expected value), 3) the variance of the expected value of the decision, 4) the outcome of the decision, 5) the signed as well as the absolute so called prediction error, i.e. the difference between the actual and expected outcome. This was done both by investigating broader patterns in the movement data as well as more specific aspects of the movement paths. The broader patterns were analyzed by investigating the link between variables of interest and types of movement paths (identified using clustering methods). More specific aspects of the movements were analyzed using measures of the paths, such as path deviation and changes in direction or measures of acceleration and deceleration (Kieslich & Henninger 2017).

In line with previous work demonstrating that decision-processes are often reflected in features of decision actions (Freeman 2018; Gallivan et al. 2018) we expected that also formally well defined, and computationally derived, variables of learning and decision processes would be linked to movement patterns, for instance with regards to the temporal or spatial aspect of the movement. We did not formulate more detailed hypotheses regarding the links between the movement patterns and each separate variable. The aim was rather to explore and investigate the nature of these patterns more broadly.

To the best of our knowledge, no study has combined computational methods of decision-making, such as RL, with mouse-tracking and a search on PsycInfo for the conjunction of the terms “reinforcement learning”, “decision making” and “mouse tracking” returned no publications (search conducted April 2023).

## Method

### Participants

For this study, 120 participants (46 males) were recruited and payed for their participation, mean age 24.5 years (SD = 5.1). Participants signed a written informed consent form before they carried out a simple probabilistic two-alternative forced choice task where mouse-tracking data were collected, in addition to decision data. Due to technical problems, data for five individuals were not collected. The final data set thus consisted of 115 participants. Participation in the study was anonymous and no personal data were collected that could be linked to individual participants. Data on age and sex was collected for those recruited to the study, but were not linked to individual behavioral data. All data were collected at the Cognition and Behavior (CoBe) Lab at Aarhus University, Denmark, in 2019. Participants were recruited via the lab’s participant pool and were required to have a Danish CPR-number (for payment reasons) and to be proficient in English.

This study was performed in line with the principles of the Declaration of Helsinki. Before data collection, the study was approved by CoBe lab’s Human Subjects Committee, but since the study was anonymous and did not pose any risk of harm to the participants, no further local ethics approval was needed. The study was not preregistered.

## Procedure and stimuli

**Fig. 1:**
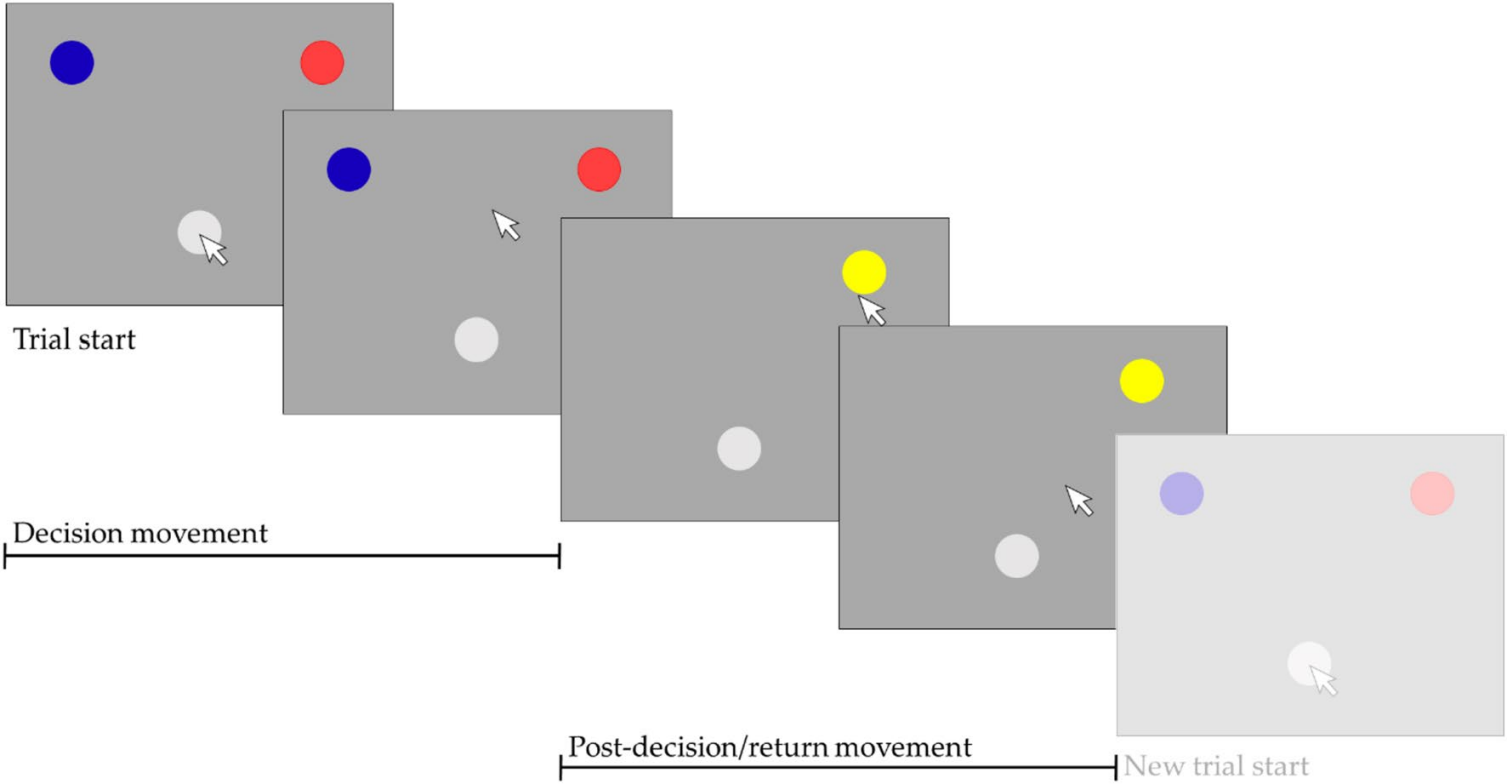
Depiction of the time course of one single (example) trial. The trial starts when the choice options are displayed, and ends when the cursor is returned to the grey circle after a choice is made. The movement made during each trial is divided into two paths: 1) the decision movement, the path from the grey circle in the bottom, the starting/return position, to the choice option (here the red circle in the top right corner), 2) the post-decision/return movement, the path following the choice back to the starting/return position. The next trial (shaded) then follows immediately without any inter-trial-interval. The choice is made when the mouse cursor crosses the edge of the choice option, upon which the choice option circle is replaced by a symbol indicating some outcome, here indicating a gain outcome, see also Fig. 1

Each participant started with a collection of 40 points and then made repeated choices between two options over a total of 360 trials separated into two blocks (Gain/Loss), to try to gain or avoid losing points (later converted to money). The choice options were associated with different probabilities (0.9/0.1) of gaining or losing a point, depending on block, such that one of the two options would always be a better option to choose than the other. A choice could thus lead to gaining a point, losing a point or no outcome, see Fig. 1. In the Gain block, the option more often leading to gaining a point was the better one to choose, whilst in the Loss block, it was the option more often leading to no outcome that was the better. In order to vary the difficulty of the task, there was a chance of 0.2 at each trial that the probabilities of gaining/losing a point associated with the two options switched. The outcome of each decision was always shown to the participant but outcome only affected their collection of points in 25% of trials (90 Exploitation trials) while participants could still learn from the remaining 75% (270 Learning trials).

Trials were divided into Exploitation and Learning trials in order to facilitate our analysis. Decision making is inherently complex, even for simple tasks. Part of this complexity lies in that fact that we often have to make decisions without knowledge of the underlying probability of outcomes. Faced with such uncertainty, we have to balance the need to gain more knowledge by exploring what will happen when we choose one option over another, with the need to optimize the outcome by exploring what we already know. This complicates decision modeling, making it hard to identify whether peoples’ choice on a trial is motivated by the need to learn (i.e. exploration) or the need to optimize outcomes (i.e. exploitation). We aimed to reduce this problem on our task, by decreasing the level of exploration in a subset of the decisions of interest (i.e. Exploitation trials) (Wilson et al. 2021), therefore making it easier to calculate (as well as interpret the meaning of) some of the variables of interest, especially choice confidence, since that might require calculating estimates of both the value of exploring as well as the value of exploiting (Dearden et al. 1998).

**Fig. 2:**
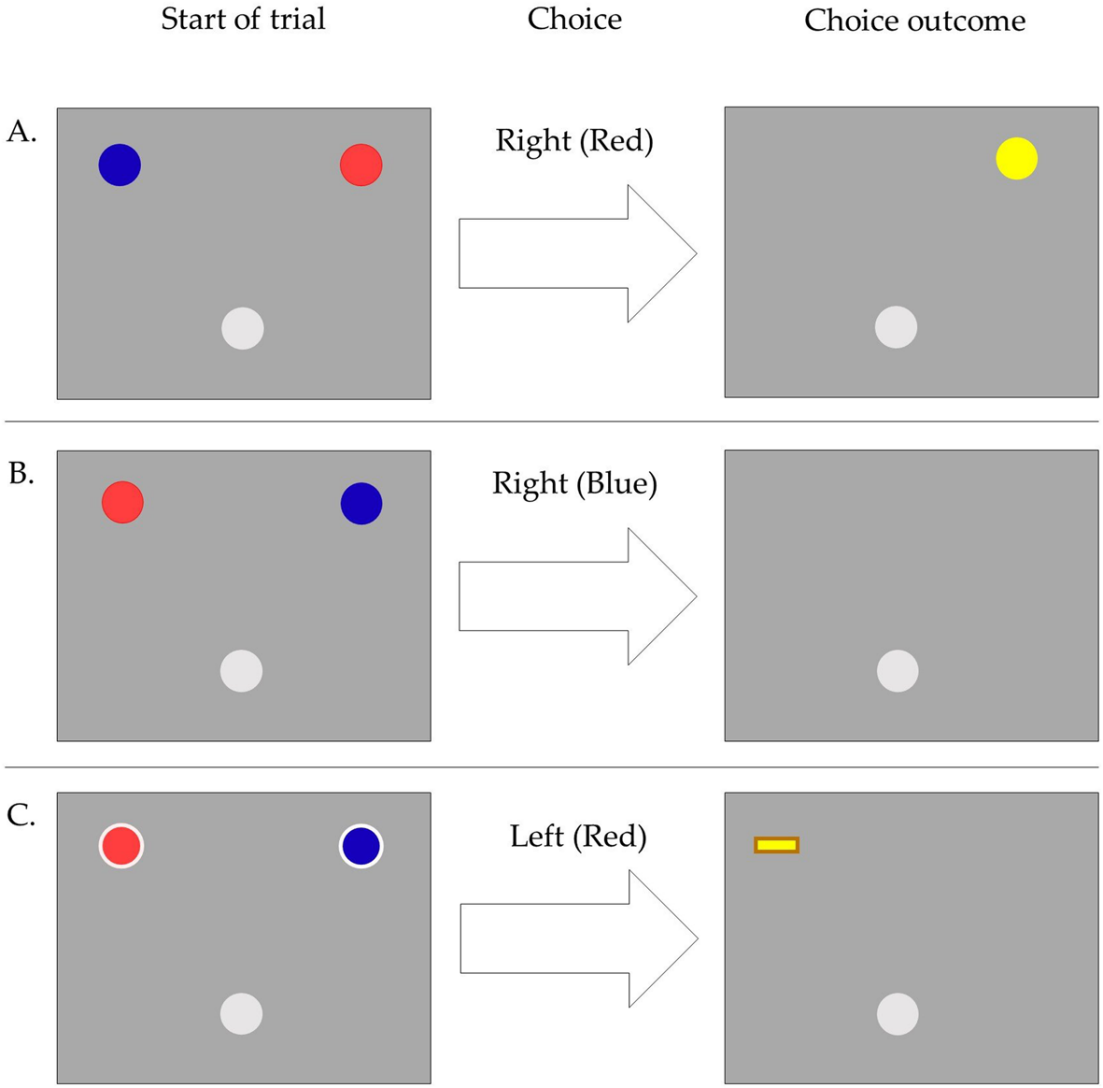
Illustration of three trial examples. The red (lighter) and blue (darker) circles in the top of panels to the left are choice options, the grey circle in the bottom of the panel is the start and return position. The yellow circle in the upmost panel on the right side indicates gain and the yellow minus in the bottom panel to the right indicates loss. The text in the middle indicates which choice the participant selected, i.e. the red choice option displayed on the right side for example A. Encircled/framed choice options and outcome indicates that the trial is an Exploitation trial. A. Learning trial, gain outcome (not counted). B. Learning trial, no outcome. C. Exploitation trial, loss outcome (counted). Note for illustrative purposes and clarity, colors and stimuli proportions are slightly different from what the participants saw. In addition, the mouse cursor is not depicted here although it was visible for the participants

The two choice options were represented on the computer screen by colored circles (red, blue) with randomized positions (left, right). Participants selected their choices by moving the mouse cursor from a starting position (a grey circle) at the bottom of the screen to the edge of one of the two colored circles, and the outcome was indicated immediately using simple symbols (a coin indicating gaining a point, a minus indicating losing a point, or nothing when no point was either gained or lost). After a choice was made, participants had to return the mouse cursor to the grey circle at the bottom of the screen, from where they had started. This movement was split into two paths. The decision path consisted of the movement of the mouse cursor from the start of the trial until a choice was made when the cursor touched or crossed the edge of one of the choice options. The post-decision/return path consisted of the movement of the mouse cursor from when the choice was made until it returned to the edge of the circle indicating the starting position. See Fig. 1 for an illustration of a single trial and how it is divided into two paths. To ensure that the distance to the two choices always was exactly the same at the beginning of each trial, the mouse cursor was automatically moved to the exact same starting point in the center of the grey circle after the return. Exploitation trials were indicated to the participant visually, by showing a bright frame or edge around both the colored circles, the choice options, as well as the outcome symbols. In order to be able to measure initiation time (i.e. the delay from the onset of the trial to the first movement) choice options were visible from the very start of the trial, although it is sometimes recommended for mouse-tracking studies that participants have to make a movement in order to see choice options (thus avoiding that participants decide first and move later) (Grage et al. 2019). In an attempt to counterbalance any tendency of the participants to decide before moving, there was no intertrial interval and thus after returning to the starting position, the next trial followed immediately. Trials were otherwise self-paced, again in order to better capture effects of speed and time. See Fig. 2 for examples of the trial layouts.

## Instructions

Before starting, participants were given written instructions on the screen. With regards to the experimental setup, they were told that the probabilities of gaining/losing points would differ between choice options and that they would change over time but they were not provided with any details regarding how this change would look. They also went through a slow step-by-step demonstration of the task setup and were able to conduct a brief practice session before the actual experiment began. We did not give participants any information before-hand regarding collection of mouse tracking data.

## Data collection and preprocessing

The experiment was programmed in Python (Van Rossum & Drake 2009) and the experiment was presented on computers with screen sizes 1536 × 864 pixels. To improve the quality of the mouse-tracking data we adjusted the speed of the mouse cursor so that it was slightly slower than normal (while making sure it was the same for all participants) and Enhance Pointer Position was disabled so that the movement of the mouse cursor on the screen corresponded to the participant’s movement of the computer mouse (Fischer & Hartmann 2014). Mouse-tracking data was preprocessed using the mousetrap package in R (Wulff et al. 2021). The data were preprocessed by aligning the mouse paths so that the starting and end, or target, position was the same for all paths, according to recommendations (Kieslich et al. 2019). This was done by first looking at how much the starting position of the recorded data deviated from the expected starting position and removing paths with abnormal data. The exclusion criterion was set to a maximum deviation of 20 pixels along either the y or x-axis. Based on this criterion, 36 of the 10,350 paths collected for the analyses were removed (approximately 0.3%). Next, according to preprocessing recommendations (Kieslich et al. 2019), paths where the selected target was the right choice option were mirrored so that all paths led to the target to the left. Paths were then divided into decision and post-decision/return paths. Finally, each path was aligned such that for each direction the paths started and ended at the exact same position; the starting position was thus set to [0,0] and the target position to [−250, 350]. The mt_measures as well as the mt_sample_entropy functions in the mousetrap package were used to extract several measures of interest from each path, what we later will refer to as action dynamics. These measures were related to maximum/minimum positions of the cursor along the x or y-axis, path deviations, directional changes, time and pausing, speed or distance as well as entropy (a measure of path complexity, see Supplementary for details). The hover threshold, which determined the threshold for when a period without movement was considered a hover, was set to 250 ms (no default value available) based on (Fernández-Fontelo et al. 2023). The flip threshold, the distance that needed to be exceeded in one direction so that a change in direction counts as a flip was set to the default of 0. The initiation threshold, the distance from the starting point that needed to be exceeded in order to calculate the initiation time, was set to the default of 0.

## Computational modeling

A computational model inspired by the reduced Bayesian RL model suggested by Nassar et al (2010) was used to model behavior. A Bayesian method allows for the computation of latent decision-making variables that are not available using most non-Bayesian RL methods, such as choice confidence and precision since it models beliefs as probability distributions. The model uses Bayesian inference to track the probability distributions, ${\text{Pr}}\left({\theta }_{t}^{i}\right)$, over the probability $\theta$ [0,1] of gaining or losing a point (+ 1 or −1, depending on block) of each choice option $i$ over trials $t$. Thus, for the two available options in each trial, the model tracks the probabilities of gaining (or losing) a point using distribution of probabilities from zero to one. At the start of each block, the model has no information about the choice options and assumes that all probabilities are equally likely (mean probability 0.5 for both choice options). When a choice is made, the outcome of that choice is used to update the belief about it such that the most recent outcome is believed to be more likely. This sampling of new information is modelled using a beta distributed likelihood function, L, that assumes zero probabilities for the extremes, 0 and 1, and with a maximum around 0.9 or 0.1 (depending on outcome):﻿

$$L = \left\{ {\begin{array}{*{20}c} {Beta(\alpha = 2,\beta = 1.1){\text{ when outcome is gain/loss}}} \\ {Beta(\alpha = 1.1,\beta = 2){\text{ when outcome is nothing}}} \\ \end{array} \begin{array}{*{20}c} {} \\ {} \\ \end{array} } \right.$$

The model includes the possibility that previous outcomes have varying impact on the tracking of $\theta$. Previous outcomes could be completely uninformative of future outcomes, and then the posterior probability distribution would always be equal to $U(\mathrm{0,1})$, i.e. all probabilities are believed to be equally likely and outcomes would have no impact on beliefs. The impact that previous information has on the posterior distribution is controlled by $\gamma$, a parameter that weights how informative previous outcomes are for future outcomes. Including $\gamma$ allows for tracking of a changing ${\theta }_{t}^{i}$, otherwise, model beliefs would capture the probabilities for each choice option across the entire block. For each trial (both Exploitation and Learning trials), probability distributions of the two choice options were updated according to:

$${\text{P}}r(\theta_{(t + 1)}^{{{\text{chosen}}}} ) \propto \gamma \times ({\text{P}}r(\theta_{(t)}^{{{\text{chosen}}}} ) \times {\text{L}}_{t}^{{{\text{outcome}}}} ) + (1 - \gamma ) \times {\text{U}}(0,1)$$

$${\text{P}}r(\theta_{(t + 1)}^{{({\text{non - chosen}})}} ) \propto \times {\text{P}}r(\theta_{t}^{{({\text{non - chosen}})}} ) + (1 - \gamma ) \times {\text{U}}(0,1)$$

Using the probability distributions ${\text{Pr}}\left( {\theta_{t}^{i} } \right)$ expected values of each choice *i* can be calculated trial-by-trial:

$${\text{EV}}_{t}^{i} = \left\{ {\begin{array}{*{20}c} {\int_{0}^{1} {\theta \Pr (\theta_{t}^{i} )d\theta {\text{ in a gain block}}} } \\ { - \int_{0}^{1} {\theta \Pr (\theta_{t}^{i} )d\theta {\text{ in a loss block}}} } \\ \end{array} } \right.$$

The probabilities of selecting each choice option (during Exploitation trials), ${\varepsilon }_{t}^{i}$, were then modelled with the Softmax decision function using the expected values, ${{\text{EV}}}_{t}^{i}$ with the temperature parameter, $T$, included as a free parameter:

$$\varepsilon_{t}^{i} = \frac{{\exp ({\text{EV}}_{t}^{i} /T)}}{{\exp ({\text{EV}}_{t}^{i} /T) + \exp ({\text{EV}}_{t}^{\neg i} /T)}}$$

The model thus included two free parameters, $\gamma$ and $T$, and was fitted to individual choice data (during Exploitation trials) using ${\varepsilon }_{t}^{i}$. Thus, although the model included learning from both types of trials, model performance was only evaluated on how well it predicted decisions during exploitation trials, where decisions are assumed to be less exploratory or noisy compared to earning trials. The model allowed for the variables of interest to be extracted from each participant and trial. We extracted six variables, three that were relevant for the decision period and three that were relevant for the post-decision period to explore the relationship to both the decision and return movement. For the decision period we decided to investigate the decision-maker’s confidence in his/her decision, the variance of the expected outcome of the choice as well as the overall expectation of the outcome of the task, what we here refer to as the estimated context. Confidence is defined as the probability, given the decision makers beliefs, that the chosen decision is the correct decision (Pouget et al. 2016). Here, confidence was calculated as the probability that the chosen option is better than the non-chosen:

$${\text{Confidence}}_{t}^{{{\text{chosen}}}} = p(\theta_{t}^{{{\text{chosen}}}} > \theta_{t}^{{({\text{non - chosen}})}} )$$

Context value was calculated as the mean expected value of the two choices:

$${\text{Context}}_{{\text{t}}} = ({\text{EV}}_{t}^{i} + {\text{EV}}_{t}^{\neg i} )/2$$

For the post-decision period we decided to investigate variables relevant for learning; the outcome of the choice, the prediction error and the absolute prediction error, as well as the resulting change in confidence for the selected choice option. The outcome was either + 1 (gain), −1 (loss) or 0 (no outcome). The prediction error, PE, was calculated as the difference between the actual and predicted outcome:

$${\text{PE}} = {\text{Outcome}} - {\text{EV}}_{t}^{{{\text{chosen}}}}$$

The absolute PE was calculated as |PE|. The change in choice confidence was calculated by comparing the confidence for the choice option before and after the choice was made:

$${\text{Confidence change}} = {\text{Confidence}}_{{\text{(t + 1)}}}^{{{\text{chosen}}}} - {\text{Confidence}}_{{\text{t}}}^{{{\text{chosen}}}}$$

Note that all these variables, apart from Outcome, depend on the beliefs of the decision maker, the participant. See Supplementary for further model details, including parameter recovery.

## Analyses

All analyses were carried out in R Statistical Software ( v.4.1.1; R Core Team 2021).

Choice data were analyzed using a logistic mixed model with Experience (the number of trials of experience of the latest set of probabilities) and Trial type (Exploitation, Learning) as predictors and Optimality (Suboptimal = 0, Optimal = 1) as the dependent variable. Optimality was defined in terms of experienced probabilities, rather than in terms of the underlying reward schedule, to ensure that the outcome measure was indicative of participants learning on the task. This meant that the “optimal” choice option was the one which had been the best to choose in the context of the most recently sampled set of probabilities. As an example, following three trials during which the red choice option was associated with a higher probability of leading to gaining a point, selecting the red choice option was determined to be optimal on the next trial, even if the probabilities had switched in the reinforcement schedule so that the red choice option was now the worse choice according to the schedule. Since this means that no choice could be considered optimal at the very first trial of each block, these trials were removed from the analyses. To facilitate model convergence we ensured that the variables were within a relatively similar range, Experience was centered at 1 and divided by 5 such that it in practice ranged from −0.2 to 8.4, but with a large proportion within the lower range. Trial type was modelled using a dummy variable (Exploitation = 0.5, Learning = −0.5).

For the mousetracking data, we analyzed the movements of the Exploitation trials only (90 trials per participant). The effects of the decision and learning variables on movement patterns were analyzed separately for each movement direction-both the path from start to target, i.e. the decision movements, and the path from target to starting position, i.e. the return movements that were carried out post-decision. During decision movements, we investigated the effect of three latent decision variables: Confidence, Variance and Context. During return movements we investigated the effect of four learning variables of interest: PE (prediction error), absolute PE, Confidence change and Outcome (win, loss, nothing). To simplify the interpretation of our analyses, all decision and learning variables that were included as independent variables were centered around zero and adjusted to be within a similar range (see Supplementary for details). The variable Context is highly correlated with whether or not the current block belongs to the gain or loss condition since the values are distributed around 0.5 during gain blocks and around −0.5 during loss blocks. Therefore, we also ran a set of models where we replaced the Context predictor with a Gain/Loss dummy predictor (gain = 0.5, loss = −0.5). Since model comparisons based on Akaike Information Criterion (AIC) showed that the models which included the computationally based Context rather than Gain/Loss better described the data in 24 of 30 cases investigating effects on movement measures we here present the results from the models including Context. For some statistical tests, the difference between the types of models was very small. The AIC for the Context-models was on average 1.79 lower (better) than for the Gain/Loss-models. As a rule of thumb, a difference of at least 2 indicate substantial support for the model with the lower AIC (Burnham & Anderson 2004) indicating that the model types are still rather similar. Conceptually, the results were very similar when comparing the two types of models, see Supplementary for more details.

We first analyzed the broad patterns of movements, by analyzing the paths using cluster analyses, such that the whole movement was considered. The cluster analyses were carried out on length-normalized mouse-tracking data, which is what is typically recommended for clustering analyses (Wulff et al. 2019) using the mt_cluster function from the mousetrap package with the number of clusters based on hierarchical clustering, but with a minimum of two clusters. We then used logistic mixed models where the independent variables were the decision and learning variables of interest and the dependent variable was the cluster, or type of path, that the associated movement belonged to. All these models included a by-subject random intercept. The alpha-level was set to 0.05.

Next, we analyzed movements by looking at certain features of, or measurements extracted from, the movements, what we here refer to as action dynamics. These action dynamics include measurements of the maximum and minimum position of each path, the deviation of the path from the direct path, directional changes of the movement, temporal aspects of the path, measurements related to speed and distance as well as a measurement of path entropy, see Table 1 for short descriptions of all measurement. The analyses were carried out by including the action dynamics measurements as dependent variables in a series of linear mixed effects models, one for each action dynamic and movement direction. Depending on the distribution of the dependent variable (e.g. continuous variables, discrete counts, bounded variables, etc.), we could not use the same type of statistical model for all variables, and thus different types of models were used to analyze the different action dynamics. In other words, all statistical modeling was conducted within the framework of the Generalized Linear Mixed Model, however the particular model used for any variable of interest was the one which was appropriate to the distribution of that variable. Dependent variables that consisted of count data were modelled with Poisson generalized mixed models using the glmer function in the lme4 package (Bates et al. 2015). Several dependent variables consisted of zero-inflated data, and were modelled with mixed hurdle lognormal models. One dependent variable was binary and was modelled with logistic mixed models. Both the mixed hurdle lognormal models and the logistic mixed models were calculated using the mixed-model function in the GLMMadaptive package (Rizopoulos 2022). The remaining dependent variables were modelled with linear mixed models using the lmer function in the lme4 package. Heavily skewed dependent variables were first log-transformed. All models included a by-subject random intercept. When needed, dependent variables were adjusted to fit the modelling procedure. For instance, since the end point of the movement always was positioned at [−250,350] (after adjusting and aligning the data), the minimum x-position consisted of negative values with an excess of values at −250, the highest possible value. To be able to include this as a dependent variable in a hurdle model, first −250 was subtracted from the values and then they were reversed, such that the excess of values was at 0 with a “tail” of positive values. See the Supplementary for details on the interpretation and transformation of each action dynamic measure. Since we did not expect the action dynamics measures to be independent we used an alpha level of 0.005 to somewhat compensate for multiple comparisons in these analyses. However, our analyses of action dynamic measures are meant to be interpreted as investigations of movement patterns rather than single independent tests.

**Table 1:** Table showing all the action dynamics measurements included in the analyses, along with a short description

Variable (unit)	Short description
Maximum/Minimum position	
Max(x-position)	Maximum position along x-axis
Min(x-position)	Minimum position along x-axis
Max(y-position)	Maximum position along y-axis
Min(y-position)	Minimum position along x-axis
Deviation	
Sign (MAD)	Signed maximum absolute deviation
log(abs(MAD))	Log of absolute maximum absolute deviation
log(MAD, time)	Log of timepoint for maximum absolute deviation
MD above	Maximum deviation above direct path
MD below	Maximum deviation below direct path
MD above, time	Timepoint for maximum deviation above direct path
MD below, time	Timepoint for maximum deviation below direct path
AD	Average deviation
AUC	Area under the curve
Directional changes	
Flips x-axis (nbr)	Number of directional changes along x-axis
Flips y-axis (nbr)	Number of directional changes along y-axis
Reversals x-axis (nbr)	Number of crossings of the x-axis
Reversals y-axis (nbr)	Number of crossings of the y-axis
Time	
RT (sec)	Response time
Initiation time (sec)	Time from trial start or decision until first movement
Idle time (sec)	Total time without movement
Hover time (sec)	Total time of hovers/pauses longer than 0.1 s
Nbr of hovers (nbr)	Total number of hovers/pauses longer than 0.1 s
Distance/Speed	
Total distance (pixels)	Total distance covered by the mouse path
Max velocity (pixels/sec)	Maximum velocity
Max velocity, time (sec)	Time at which maximum velocity occurred
Max acceleration (pixels/sec^2^)	Maximum acceleration
Max acceleration, time (sec)	Time at which maximum acceleration occurred
Min acceleration (pixels/sec^2^)	Minimum acceleration
Min acceleration, time (sec)	Time at which minimum acceleration occurred
Entropy	
Entropy (-)	Measure of path complexity

## Results

### Learning and performance

**Fig. 3:**
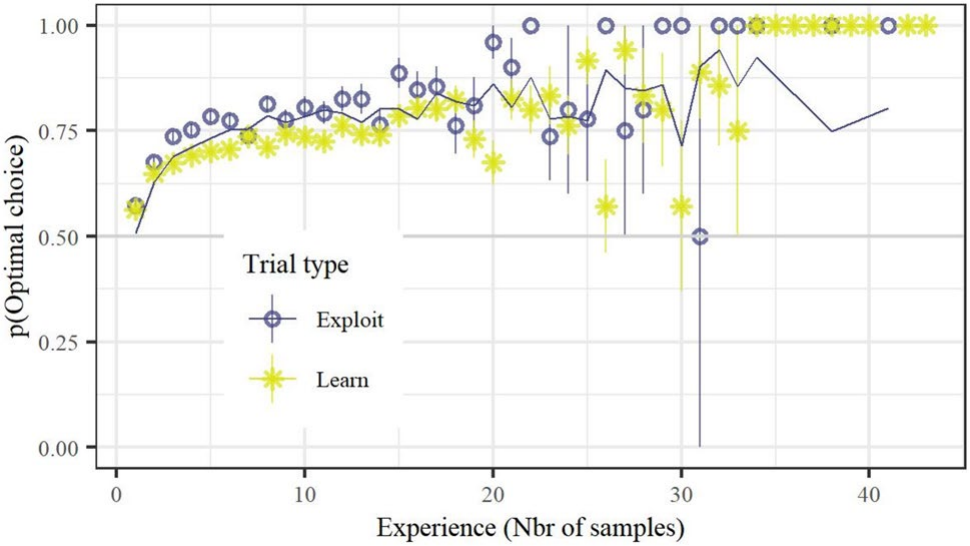
Performance as a function of experience and trial type. Points indicate data and lines show the mean predicted behavior of all individuals based on the computational model, error bars indicate standard error. The figure clearly shows that decisions are less exploratory during Exploitation trials in addition to illustrating that the computational model is able to reproduce behavior rather well. (See Supplementary for a visualization of predicted behavior per participant)

Participants learned the task well with increased experience (Experience: ***Χ***^*2*^(1) = 125.29, Estimate = 0.54, SE = 0.049, *z* = 11.19, *p* < 0.001). As intended, participants also appeared to explore less during Exploitation trials, as evident by a higher proportion of optimal choices and a steeper learning curve during those trials compared to learning trials (Trial type: ***Χ***^*2*^(1) = 4.62, Estimate = 0.088, SE = 0.041, *z* = 2.15, *p* = 0.032; Trial type × Experience: ***Χ***^*2*^(1) = 13.02, Estimate = 0.22, SE = 0.060, *z* = 3.61, *p* < 0.001), see also Fig. 3. An analysis of the distribution of the fitted parameters of the computational model showed that the parameter guiding the influence of previous outcomes, $\gamma$, was heavily skewed towards 1, with a median value of approximately 0.995, while the temperature parameter, *T*, was heavily skewed towards the lower threshold 0.01, with a median of approximately 0.054, indicating a low degree of exploration/noise for the decisions made during exploitation trials.

## Decision movement

### Broad patterns

**Fig. 4:**
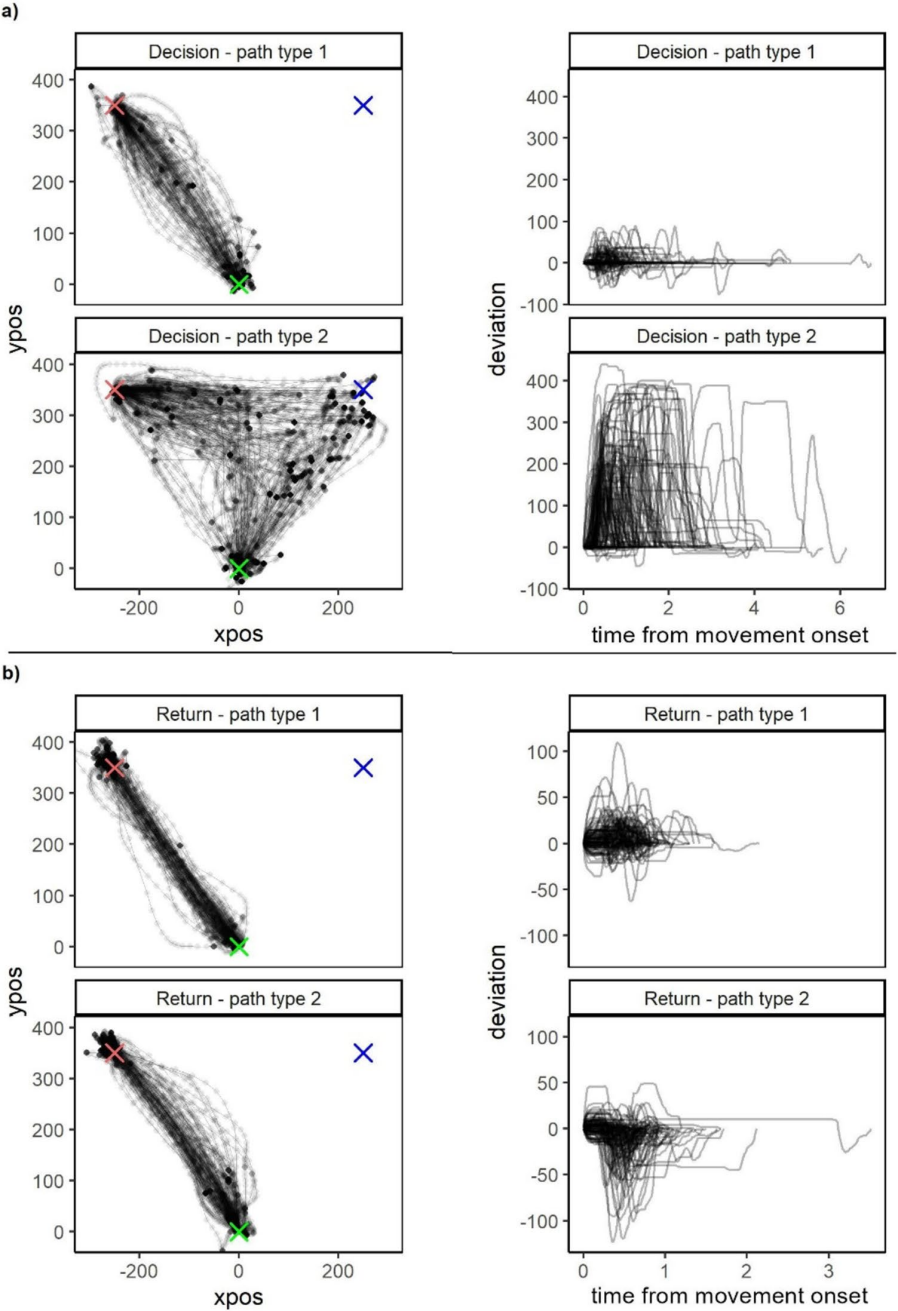
Illustration of different type of paths, based on cluster analyses of length normalized data from a) decision and b) return (post-decision) movements. For each path type, a random subset of 100 paths are plotted in an xy-plane to the left. This gives a representation of movement displacement in the two spatial axes on the selected paths. On the right, the same movement paths are represented, but as deviations from the shortest path to target over time from onset. This gives a representation of the time course of the movement variance or error for the selected paths. This allows us to see both the full spatial and temporal differences between the two path types. In the left column, the green cross in the bottom represents the starting point, the red (lighter) cross to the left the target (selected option) and the blue (darker) cross to the right the non-selected option. Note that the data is preprocessed so that the target is always shown to the left even when the participant selected the right option and such that the starting position and target position is always on the exact same spot. Note that the scale of the y-axis in the plots to the right differs between a) and b)

Clustering of length-normalized decision data resulted in two clusters of movement paths, see Fig. 4a. Most paths (89%) were classified as belonging to path type 1 which can be described as a more direct path, while the remaining paths (11%) were classified as belonging to path type 2 which typically included deviations towards the non-chosen option as well as pauses or hovers. Analyses using logistic mixed modeling showed a link between the latent decision variables and type of path such that higher Confidence and higher Context were associated with a higher probability of that the path was a more direct path, belonging to path type 1 in Fig. 4a (Confidence: ***Χ***^*2*^(1) = 21.96, Estimate = −3.07, SE = 0.66, *z* = −4.69, *p* < 0.001; Context: ***Χ***^*2*^(1) = 6.21, Estimate = −0.30, SE = 0.12, *z* = −2.49, *p* = 0.013). There was no effect of Variance on type of path (*p* = 0.60).

### Action dynamics

The analyses of the action dynamics related to the decision movements showed effects of both Confidence and Context on nearly all types of action dynamic measures, see Table 2. There were also some effects of Variance on some of the action dynamic measures related to timing. The effects of Confidence and Context typically appeared to follow similar patterns. Effects on the measures of the maximum and minimum position along the y- or x-axis as well as on the measures related to deviation from the straightest path show that both high Confidence and high Context were related to movement paths that deviated less from the straightest path-for instance less extreme maximum and minimum positions, smaller maximum absolute deviation (MAD) that occurs earlier in time, smaller deviations above the straightest path and smaller average deviations. Effect on measures related to directional changes show that both high Confidence and high Context were related to fewer directional changes, along both the x and y-axis. Effects on time-related measures show that both high Confidence and high Context were related to decision movements with shorter response times (RT), less idle and hover time and fewer hovers or pauses. Effects on distance and speed-related measures show that both high Confidence and high Context were for instance related to shorter paths with the greatest changes in speed at the beginning of the movement. The effects of Variance were fewer and typically in the opposite direction from the effects of confidence and context. For instance, the timepoint at which the maximum absolute deviation (MAD) occurred later when Variance was higher, while it occurred earlier when Confidence or Context was higher. Higher variance was also associated with longer response times (RT), in contrast to higher values of Confidence or Context that were associated with shorter response times.

**Table 2:**
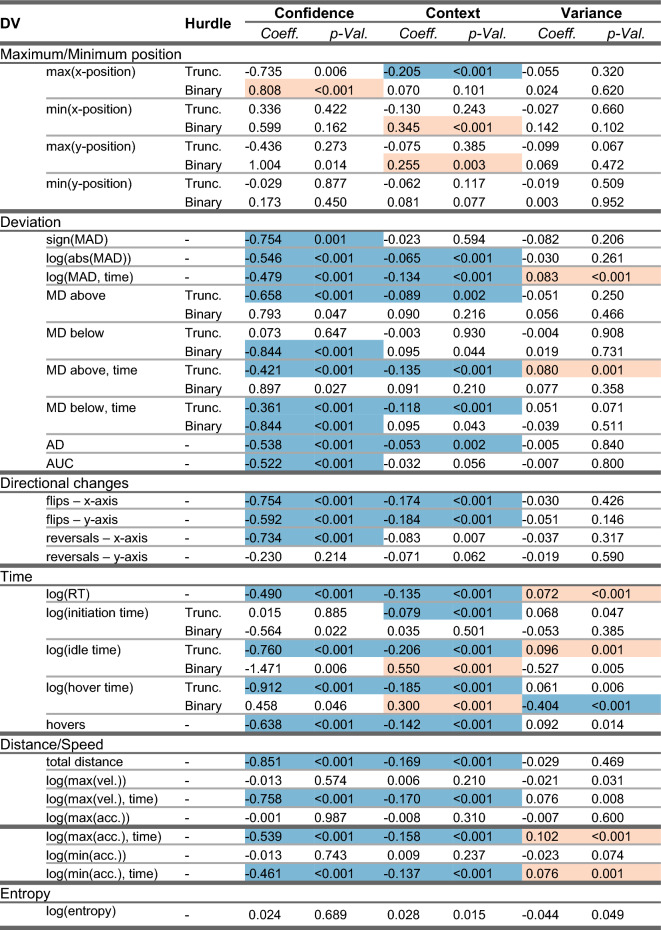
Coefficients and *p*-values for the three variables of interest relevant for the decision period. Significant values (here, *p* < 0.005) are indicated with a shaded background, red (lighter shade) when the coefficient is positive and blue (darker shade) when it is negative. Results from hurdle models are presented as two separate model parts, the binary part and the truncated part

## Post-decision/return movement

### Broad patterns

Clustering of length-normalized return data resulted in two clusters of movement paths, see Fig. 4b. Both path types are rather straight, roughly half of the paths (40%) were classified as belonging to path type 1 where deviations typically drift away from the non-chosen target side, while the remaining paths (60%) were classified as belonging to path type 2 which typically included deviations towards the non-chosen target side. Analyses using logistic mixed modeling showed an effect of the prediction error (PE) such that higher PE was associated with a higher probability of a path belonging to path type 1 in Fig. 4b (***Χ***^*2*^(1) = 7.26, Estimate = −0.28, SE = 0.10, *z* = −2.70, *p* = 0.007). Although the qualitative differences between the clusters appear small, path type 2 can be described as being slightly more bent towards the right (non-target) side compared to path type 1. We saw no effects of the absolute prediction error, abs(PE), (*p* = 0.54), Outcome (*p* = 0.59) or Change in Confidence (*p* = 0.11) on type of path.

### Action dynamics

The analyses of the action dynamics related to the return movements showed effects of three of the latent variables of interest, PE, abs(PE) and outcome, although to a lesser degree compared to the effects of latent variables on decision movement, see Table 3. Higher PE was related to smaller maximum y-position, i.e. when outcome was more positive than expected, movements upwards on the screen were more restricted. Most effects were seen for abs(PE), essentially a measure of surprise, most clearly for action dynamic measures related to time and speed. Higher values of abs(PE) were for instance related to longer response times (RT), longer idle time and a larger number of hovers. Larger values of abs(PE) was also related to higher number of crossings of the x and y-axes (reversals) and a larger likelihood of a movement that at some point was closer to the side of screen belonging to the non-chosen choice option. Further, larger abs(PE) were related to movements where the maximum and minimum acceleration and maximum velocity occurred later during the movement while also associated with reduced velocity and greater decreases in velocity (minimum acceleration). Outcome had some effects on the movement measures such that positive outcomes were associated with greater movements upwards on the screen (maximum y-position) and larger area under the curve (AUC) indicating larger deviation from the straightest path. Change in Confidence did not have any effect on movement measures.

**Table 3:**
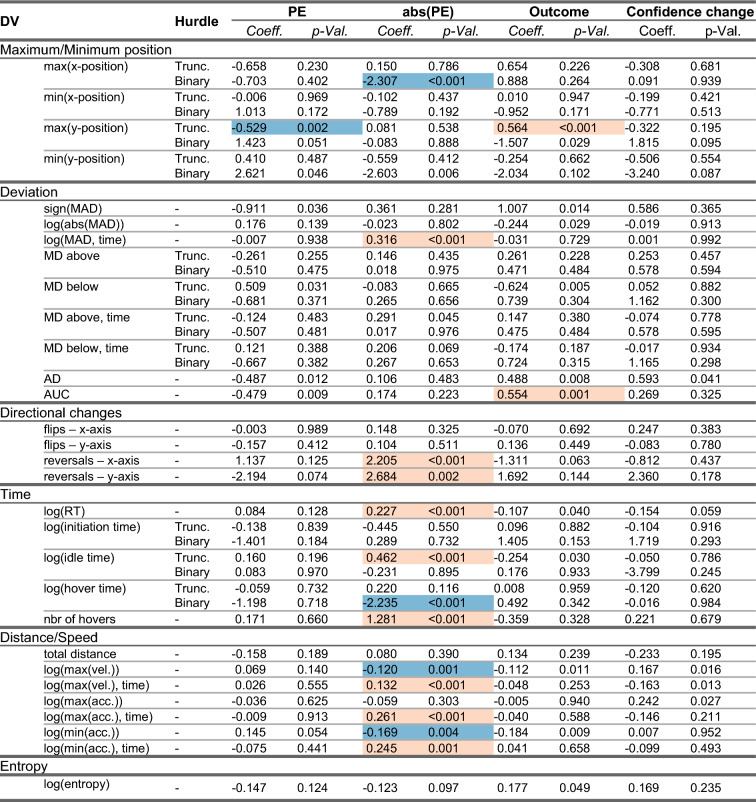
Coefficients and p-values for the four variables of interest relevant for the post-decision/return period. Significant values (here, *p* < 0.005) are indicated with a shaded background, red (lighter shade) when the coefficient is positive and blue (darker shade) when it is negative. Results from hurdle models are presented as two separate model parts, the binary part and the truncated part

## Conclusion

To conclude, we show that variables related to decision making and learning can be linked to features of the movement patterns associated with both the decision and post-decision action. The results were most pronounced during the decision action where both confidence and context were clearly linked to what we could refer to as the directness of the movement as well as time-related aspects such as speed and pausing. The effects were less pronounced during the post-decision action where we mainly saw effects of the absolute Prediction error on aspects related to time and speed. Given that participants, after making a decision, simply returned to the starting point, and that there was no alternative target to move to, it is not unexpected that the effects during the post-decision movement are less pronounced.

## Discussion

Decisions have often been thought of as a planning process that takes place before the selected decision is executed, typically by making some action. More recent research has however demonstrated that the selection and execution of an action are not always clearly separable (Cisek 2007; Freeman 2018), and that the decision-making process is often reflected in for instance the movements needed to carry out an action (Gallivan et al. 2018).

In this study, we wanted to explore if and how formally well-defined concepts related to decision making and learning could be linked to movement patterns during decision actions. As predicted, our results clearly show that several important variables linked to decision making and learning, mainly Confidence, Context, Variance and the absolute Prediction Error, were correlated with movement paths, especially during decision making. Interestingly, the clearest effects were seen for Confidence and Context, two variables that could be of great interest to someone observing the decision maker (Bahrami et al. 2010; Griffin 2004; Lindström et al. 2016; Marshall et al. 2017), for instance to learn themselves to make decisions, for example by to a larger extent copying decisions that appear more, rather than less, confident, or by avoiding or being more precautious in negative or dangerous contexts. The effects of these two variables seem to coincide to a large extent, such that the movement patterns related to high confidence are similar to those related to a highly valued Context (i.e. the effects go in the same direction) making it harder for an observer (human or machine) to distinguish between them. However, some differences can be seen in the result patterns. The most clear example is that Context, but not Confidence, correlated with the initiation time, the time it took to initiate the first movement. Here, higher Context value was linked to faster initiation, results in line with the work by Shadmehr and colleagues (Shadmehr & Ahmed 2021) showing that movements are more vigorous when the goal of the movement is more valued. Considering that low Context values are seen in the blocks where participants lose points, another suggestion is that this effect can be explained by the phenomena of behavioral inhibition, i.e. slower initiation, seen in threatening environments (Bach 2015). At this point, it is unclear how robust these differences in result patterns are. One limitation of the study presented here is that we have focused on movements during decisions that are not (to any large extent) exploratory. In a real-world setting, knowing whether or not a decision is exploratory or not could be very valuable from the point of view of an observer. Although our results show that the participants indeed did explore less, or at least performed better, during Exploitation trials, we don’t believe that the current paradigm would work well for investigating the differences in movements patterns between decisions that are either exploratory or exploitative. Such a paradigm would need to be designed to answer this particular question, for instance by making sure that exploratory trials are more purely exploratory than we believe they are in our paradigm. This could however be an interesting question for future studies. Further, previous studies have shown that when reporting decision confidence, people appear to vary in how they estimate confidence–for some individuals, reported confidence seem to reflect perceived uncertainties in the decision that are unrelated to the probability of being correct (Navajas et al. 2017). It could be an interesting possibility for future investigations to look deeper into the link between movement patterns and verbal reports of confidence to see if there are similar individual patterns in movement as there appear to be in verbal reports. Another limitation of the study is that we do not know how well our findings would generalize between individuals. We do for instance not know whether or not our results would apply equally well to men or women, old or young or if there are culturally dependent differences in these movement patterns. We would for instance expect longer response times in an older population (Theisen et al. 2021) although we currently see no reason to expect differences in how decision and learning processes are reflected in movements. We have also studied a very restricted form of movement, that of moving a computer mouse to make a decision in a simple computerized task. It is currently unclear how well these patterns would transfer to a more realistic setting, were movements are typically three-dimensional.

We believe that the results presented here can be of great interest for several disciplines, such as researchers working on social learning or human–machine/human–robot interactions. In the latter research field there is great interest in the possibility for a machine, artificial intelligence or robot, to interpret, or possibly even reproduce, subtle human cues that can guide cooperation or otherwise aid interactions (Wagner et al. 2011). Such research on “social signal processing” typically use text, pictures and video to detect emotions or action plans (Chen et al. 2019; Li et al. 2018; Liu et al. 2021) and the findings we present here might be of interest within this context. We further hope that our work can broaden the range of methods used to investigate human, and non-human, decision making.

### Supplementary Information

Below is the link to the electronic supplementary material.

Supplementary file1 (DOCX 748 KB)

## Data Availability

The datasets generated and analyzed during the current study are available at the OSF repository, https://osf.io/hr5g6/?view_only=4071b6d15664416bb59e935165d496dc.
