# Supplementary material for: The expression of decision and learning variables in movement patterns related to decision actions
Source: Exp Brain Res. 2024 Mar 29;242(6):1311–25. doi: 10.1007/s00221-024-06805-y (PMC11108959; doi:10.1007/s00221-024-06805-y)
Supplement: Supplementary file 1 — Supplementary file1 (DOCX 748 KB) [file 221_2024_6805_MOESM1_ESM.docx]

# Supplementary material

**Submission - Experimental Brain Research**

**The Expression if Decision and Learning Variables in Movement Patterns Related to Decision Actions**

Ida Selbing^1,3^ and Joshua Skewes^2,3^

^1^ Division of Psychology, Karolinska Institutet, Solna, Sweden

^2^ Department for Linguistics, Cognitive Science, and Semiotics, Aarhus University, Aarhus, Denmark

^3^ Interacting Minds Centre, Aarhus University, Aarhus, Denmark

**Corresponding author:** Ida Selbing, idaselbing@gmail.com


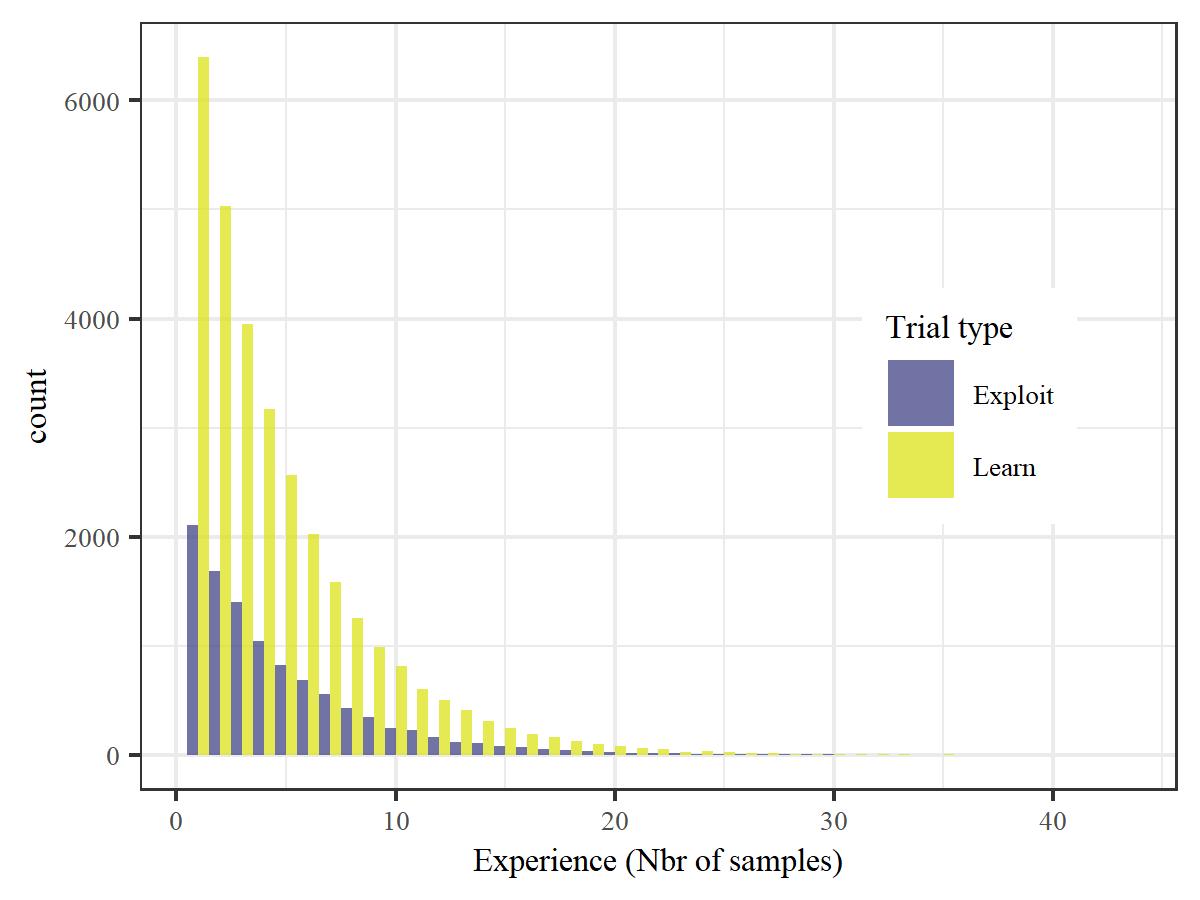


SI. Fig 1. Illustration of the distribution of the number of trials of each trial type over time/experience.

Model details

The model was implemented using grid approximation. Confidence and Variance were calculated using a bootstrap method. Model fitting was carried out using R Statistical Software (v.4.1.1; R Core Team, 2021), $\gamma$ was constrained within the interval [0,1] and $T$ was constrained within the interval [0.01, 5]. Parameters were fitted to each participant over all Exploitation trials by minimizing the negative log likelihood, using the mle2 function in the bbmle package (Bolker & R Development Core Team, 2021) with the optim optimization function and the BFGS optimization method. To avoid local minima, each set of parameters were fitted 10 times with randomized initial parameters after which the parameters that generated the best fit were selected.


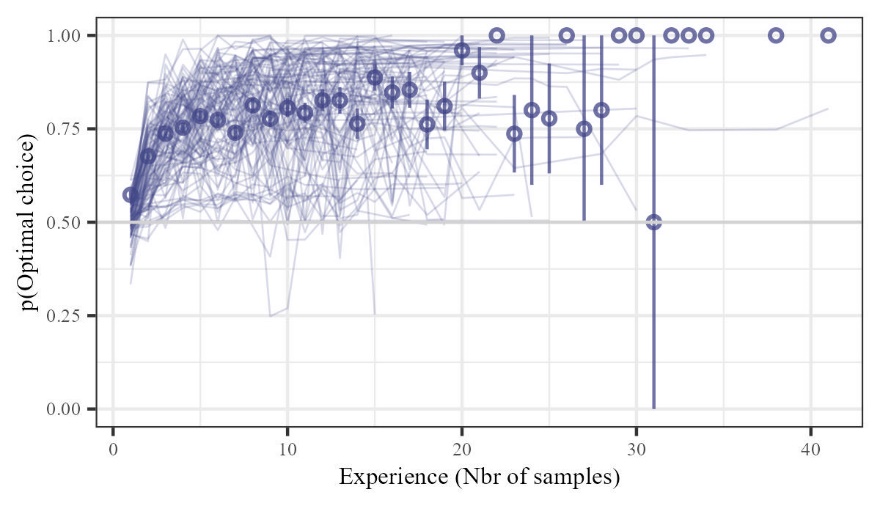


SI. Fig 2. Performance during the exploit trials as a function of experience. Points show mean performance from the collected data (error bars indicate standard error) and lines show the mean predicted behavior per participant based on the computational model. For clarity, choices during learning trials are not included.


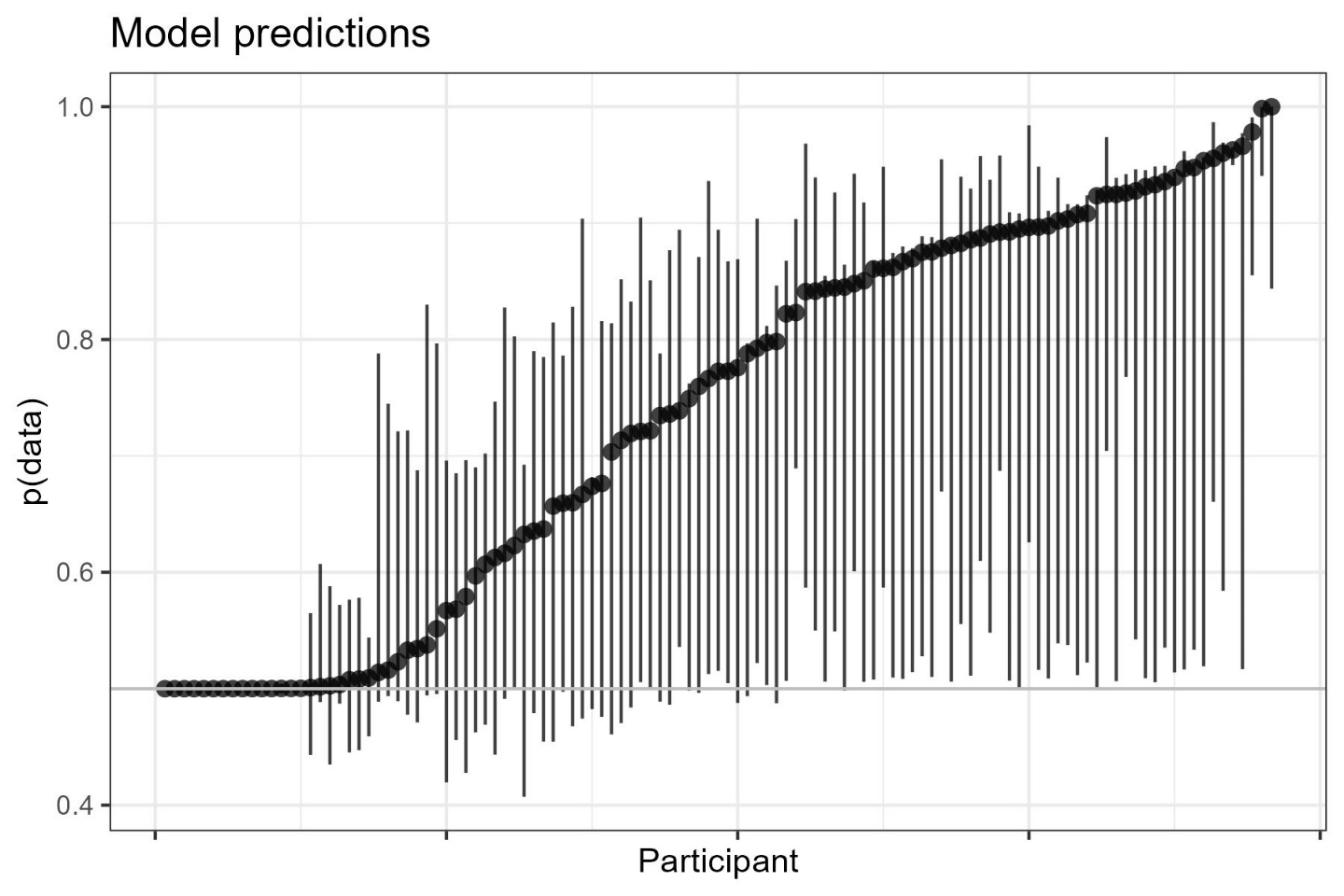


SI. Fig 3. Model performance in terms of how well the model predicted the data for all trials for each participant. Points indicate the median probability and the lines indicate the intervals within which 50% of the probabilities fall. For p(data), a value of 0.5 indicates chance while 1 indicates perfect prediction.

Model recovery

We performed parameter recovery where we simulated behavior based on parameters taken from the distribution of fitted parameters (outcome impact: heavily skewed towards 1, median value = 0.993; inverse parameter: heavily skewed towards 0, median value = 0.052). Since our model did not model decision-making during learning trials we decided to simulate data for our parameter recovery in two ways: in one we assumed that the decisions made and outcomes received during learning trials was the same as in the collected data (actual learning decisions) and in the other we assumed that the decisions made during learning trials were random and that outcomes were based on the underlying outcome probability (random learning decisions). Importantly, note that the model was only evaluated on how well it predicted the decisions made during the exploit trials. All in all, we thus carried out parameter recovery on two sets of simulated data, both based on the data we had collected, using the same underlying outcome probabilities as in the real experiment.

Since the study’s main focus lies in the variables associated with the learning and decision processes rather than the fitted parameters themselves we also investigated how well we could recover these values. Comparisons between simulated and fitted values were carried out using Spearman correlation, se SI Table 1. Spearman’s ρ. Following the recommendations of Dancey and Reidy (2007) in naming the strength of the correlation we have moderate to strong correlations for the model parameters but the parameter recovery was better for the variables of interest with typically strong correlations. To visualize the correlations, we further plotted the parameters used for simulating behavior against the recovered parameters, for both the actual and the random decisions as learning experience, see SI Fig. 5.

|  |  | Spearman’s ρ | |
| --- | --- | --- | --- |
|  |  | Actual learning decisions | Random learning decisions |
| Parameters | | |  |
|  | Outcome impact | 0.524 | 0.703 |
|  | Inverse temperature | 0.578 | 0.530 |
| Variables of interest | | |  |
|  | Confidence | 0.750 | 0.870 |
|  | Context | 0.954 | 0.978 |
|  | Variance | 0.489 | 0.698 |
|  | Prediction error | 0.954 | 0.974 |
|  | Abs(prediction error) | 0.837 | 0.898 |
|  | Confidence change | 0.724 | 0.803 |

SI Table 1. Correlations between simulated and recovered model parameters as well as simulated and recovered variables of interest. Recovery was carried out using two assumptions of decisions during learning trials.


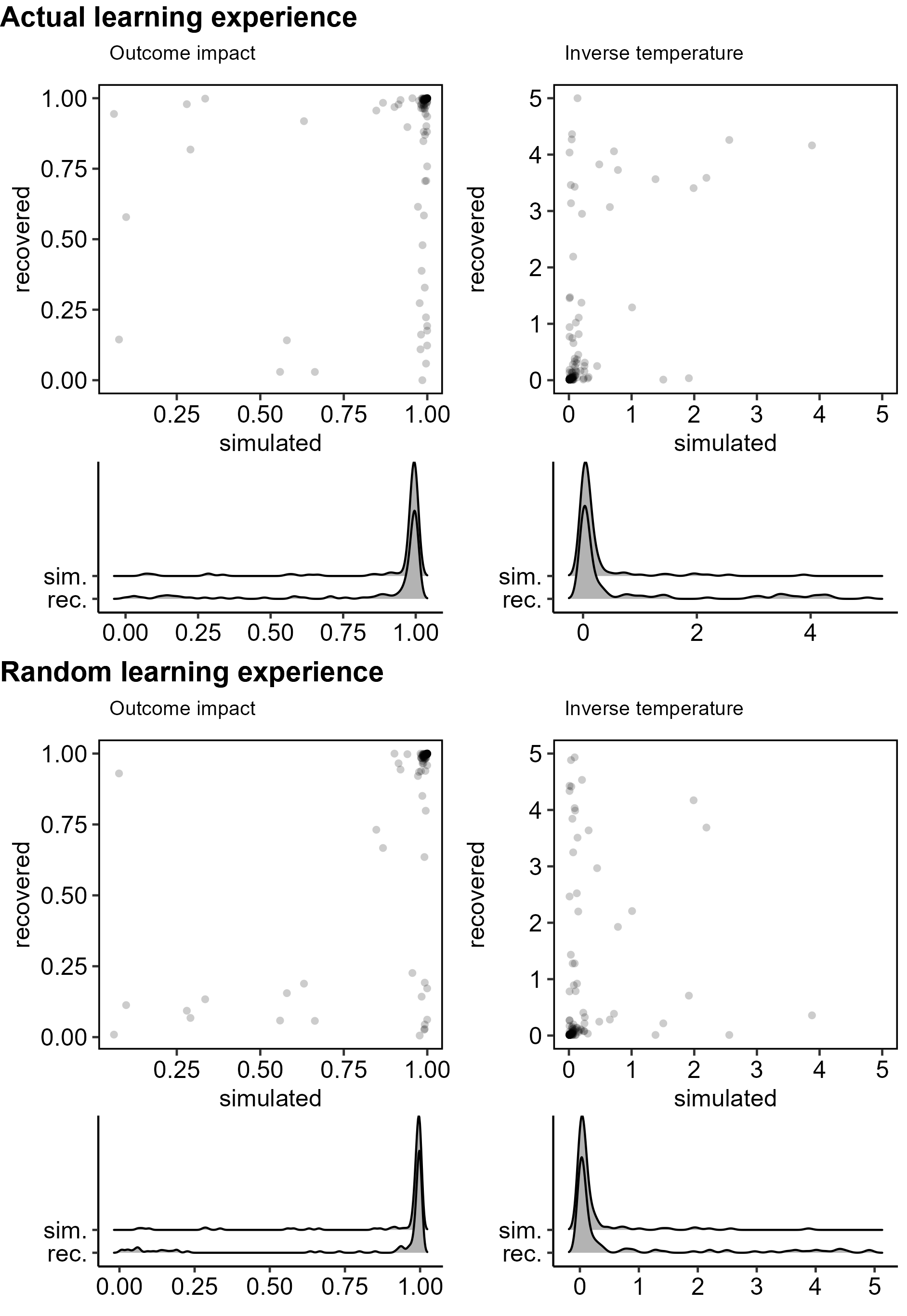


SI Fig.4 Fitted/simulated parameters plotted against recovered parameters in the simulations using both the actual and random learning experiences. The distributions of the parameters are plotted at the bottom.

Preprocessing of independent variables of interest before analyses

IVs were centered and/or scaled to be within similar ranges

Decision

Confidence was centered at 0.5, range was [-0.5, 0.5]

Context remained unchanged, range was [-0.75, 0.72]

Gain/Loss (see SI Table1) was modelled as a dummy variable, Loss = -0.5, Gain = 0.5

Variance was mean centered and normalized, range [-4.7, 0.7]

Post-decision/Return

PE remained unchanged, range was [-0.8, 0.8]

Abs(PE) was centered at 0.5, range was [-0.33, 0.31]

Outcome remained unchanged [loss = -1, nothing = 0, gain = 1]

Confidence change remained unchanged, range was [0.22, 0.24]

Transformation of dependent variables and model type used

|  | Original measure (unit) | Transformation *f(x)* | DV used in models (unit) | Model type |  |
| --- | --- | --- | --- | --- | --- |
| Maximum/Minimum position | |  |  |  | |
|  | Max x-position (pixels) | No change | Max(x-position) (pixels) | Hurdle |  |
|  | Min x-position (pixels) | 250-x | min(x-position) (pixels) | Hurdle |  |
|  | Max y-position (pixels) | x-350 | max(y-position) (pixels) | Hurdle |  |
|  | Min y-position (pixels) | -x | Min(y-position) (pixels) | Hurdle |  |
| Deviation | |  |  |  | |
|  | MAD (pixels) | x > 0 | Sign (MAD) | Logit |  |
|  | MAD (pixels) | Log(\|x\|) | log(abs(MAD)) | Gaussian |  |
|  | MAD time (sec) | Log(x) | log(MAD, time) | Gaussian |  |
|  | MD above (pixels) | No change | MD above | Hurdle |  |
|  | MD below (pixels) | -x | MD below | Hurdle |  |
|  | MD above, time (sec) | Log(x) | MD above, time | Hurdle |  |
|  | MD below, time (sec) | Log(x) | MD below, time | Hurdle |  |
|  | AD (pixels) | (x/RMS(*X*))^*^ | AD | Gaussian |  |
|  | AUC (pixels^2^) | (x/RMS(*X*))^*^ | AUC | Gaussian |  |
| Directional changes | |  |  |  | |
|  | Flips x-axis (nbr) | No change | flips x-axis (nbr) | Poisson |  |
|  | Flips y-axis (nbr) | No change | flips y-axis (nbr) | Poisson |  |
|  | Reversals x-axis (nbr) | No change | reversals x-axis (nbr) | Poisson |  |
|  | Reversals y-axis (nbr) | No change | reversals y-axis (nbr) | Poisson |  |
| Time | |  |  |  | |
|  | RT (sec) | Log(x) | log(RT) | Gaussian |  |
|  | Initiation time (sec) | No change | log(initiation time) | Hurdle |  |
|  | Idle time (sec) | No change | log(idle time) | Hurdle |  |
|  | Hover time (sec) | No change | log(hover time) | Hurdle |  |
|  | Nbr of hovers (nbr) | No change | nbr of hovers (nbr) | Poisson |  |
| Distance/Speed | |  |  |  | |
|  | Total distance (pixels) | Log(x-sqrt(250^2 + 350^2)) | total distance | Gaussian |  |
|  | Max velocity (pixels/sec) | Log(x) | log(max(vel.)) | Gaussian |  |
|  | Max velocity, time (sec) | No change | log(max(vel.), time) | Gaussian |  |
|  | Max acceleration (pixels/sec^2^) | Log(x) | log(max(acc.)) | Gaussian |  |
|  | Max acceleration, time (sec) | Log(x) | log(max(acc.), time) | Gaussian |  |
|  | Min acceleration (pixels/sec^2^) | Log(-x) | log(min(acc.)) | Gaussian |  |
|  | Min acceleration, time (sec) | Log(x) | log(min(acc.), time) | Gaussian |  |
| Entropy | |  |  |  | |
|  | Entropy (-) | Log(x) | log(entropy) | Gaussian |  |

SI Table 2.

^*^ scaled by root-mean-square (RMS)

Description and interpretation of dependent variables:

- Maximum and minimum x/y-positions denote the maximum and minimum position along the x- and y-axis respectively. Preprocessing results in zero-inflated distributions that can be modelled with hurdle models. Positive effects of the binary part should be interpreted as increased likeli-hood of a zero (i.e. the least extreme maximum/minimum position). Positive effects on the truncated part of the model should be interpreted as more extreme maximum/minimum positions.
- MAD denotes the signed maximum absolute deviation from the direct path. This variable is split into two: sign(MAD) denotes whether the maximum absolute deviation occurred above (positive) or below (negative) the direct path from start to target and log(abs(MAD)) is a measure of the absolute deviation. MAD time denotes the time at which MAD occurred.
- MD above/below denotes the maximum deviation above/below the direct path. In the original measure MD below is negative. MD below is reversed during preprocessing so that the both distributions are positive and zero inflated. Positive effects of the binary part should be interpreted as increased likelihood of a zero (the smallest maximum deviation) and positive effects of the truncated part should be interpreted as more extreme deviations. MD above/below time denotes the time at which MD above/below occurred.
- AD is the average deviation from the direct path. Positive effects should be interpreted as deviations that are more above the direct path.
- AUC is the area under the curve. Areas below the direct path are subtracted from areas above the direct path. Positive effects should be interpreted as having larger areas above the direct path.
- Flips x/y-axis denotes the number of directional changes along the x/y-axis. The flip threshold, the distance needed to be exceeded to count as a flip, was set to 0 (the default).
- Reversals x/y-axis denotes the number of crossings of the x/y-axis.
- RT is the response time, i.e. the time from the starting point to the target or vice versa (depending on path direction).
- Initiation time is the time it takes from the start of trial-phase to the point at which the first movement is made. The movement had to exceed the initiation threshold which was set to 0 (the default).
- Idle time is the total time without movement during the entire path.
- Hover time is the total time of periods, exceeding a hover threshold of 0.1 s, without movement across the path.
- Nbr of hovers is the number of periods without movement across the path.
- Total distance covered by the mouse path. The distance is preprocessed by log-trans-formation after subtracting the shortest distance from the start to the target position.
- Max velocity is the maximum velocity and max velocity time was the time point during the movement with maximum velocity. (Minimum velocity and minimum velocity time were not included in the analyses because they were both always 0.)
- Max/Min acceleration denotes the maximum and minimum acceleration, i.e. the greatest increase in velocity and the greatest decrease in velocity, slowing down. Min acceleration is thus typically a negative value. Max/min acceleration time denotes the time at which these measures occurred.
- Entropy is a measure of the path complexity. Here, it is computed by comparing windows of fixed size across all recorded positions in line with recommendations by Hehman et al (2015).

Model results using Gain/Loss as independent variable instead of Context

| **DV** |  | **Hurdle** | **Confidence** | | **GainLoss** | | **Variance** | |
| --- | --- | --- | --- | --- | --- | --- | --- | --- |
|  |  |  | *Coeff.* | *p-Val.* | *Coeff.* | *p-Val.* | *Coeff.* | *p-Val.* |
| Maximum/Minimum position | | | | | | | | |
|  | max(x-position) | Trunc. | -0.751 | 0.005 | -0.183 | 0.001 | -0.055 | 0.319 |
|  |  | Binary | 0.820 | <0.001 | 0.065 | 0.129 | 0.025 | 0.608 |
|  | min(x-position) | Trunc. | 0.334 | 0.424 | -0.143 | 0.201 | -0.028 | 0.655 |
|  |  | Binary | 0.608 | 0.156 | 0.337 | 0.001 | 0.142 | 0.102 |
|  | max(y-position) | Trunc. | -0.440 | 0.268 | -0.079 | 0.366 | -0.099 | 0.066 |
|  |  | Binary | 1.012 | 0.014 | 0.247 | 0.004 | 0.069 | 0.476 |
|  | min(y-position) | Trunc. | -0.033 | 0.862 | -0.066 | 0.098 | -0.019 | 0.502 |
|  |  | Binary | 0.180 | 0.432 | 0.087 | 0.059 | 0.003 | 0.946 |
| Deviation | | | | | | | | |
|  | sign(MAD) | - | -0.755 | 0.001 | -0.021 | 0.630 | -0.082 | 0.207 |
|  | log(abs(MAD) | - | -0.548 | <0.001 | -0.059 | <0.001 | -0.030 | 0.271 |
|  | log(MAD, time) | - | -0.484 | <0.001 | -0.133 | <0.001 | 0.085 | <0.001 |
|  | MD above | Trunc. | -0.664 | <0.001 | -0.082 | 0.005 | -0.050 | 0.261 |
|  |  | Binary | 0.848 | 0.034 | 0.085 | 0.246 | 0.057 | 0.460 |
|  | MD below | Trunc. | 0.072 | 0.651 | -0.001 | 0.963 | -0.004 | 0.905 |
|  |  | Binary | -0.842 | <0.001 | 0.105 | 0.026 | 0.020 | 0.727 |
|  | MD above, time | Trunc. | -0.425 | <0.001 | -0.134 | <0.001 | 0.081 | <0.001 |
|  |  | Binary | 0.744 | 0.064 | 0.083 | 0.253 | 0.069 | 0.414 |
|  | MD below, time | Trunc. | -0.364 | <0.001 | -0.117 | <0.001 | 0.053 | 0.064 |
|  |  | Binary | -0.841 | <0.001 | 0.106 | 0.024 | -0.039 | 0.506 |
|  | AD | - | -0.540 | <0.001 | -0.047 | 0.006 | -0.005 | 0.853 |
|  | AUC | - | -0.523 | <0.001 | -0.026 | 0.118 | -0.006 | 0.808 |
| Directional changes | | | | | | | | |
|  | flips – x-axis | - | -0.760 | <0.001 | -0.166 | <0.001 | -0.028 | 0.453 |
|  | flips – y-axis | - | -0.599 | <0.001 | -0.181 | <0.001 | -0.050 | 0.155 |
|  | reversals – x-axis | - | -0.737 | <0.001 | -0.077 | 0.012 | -0.037 | 0.320 |
|  | reversals – y-axis | - | -0.233 | 0.209 | -0.076 | 0.046 | -0.019 | 0.590 |
| Time | | | | | | | | |
|  | log(RT) | - | -0.495 | <0.001 | -0.133 | <0.001 | 0.074 | <0.001 |
|  | log(initiation time) | Trunc. | 0.012 | 0.907 | -0.079 | <0.001 | 0.069 | 0.044 |
|  |  | Binary | -0.563 | 0.022 | 0.036 | 0.489 | -0.054 | 0.374 |
|  | log(idle time) | Trunc. | -0.766 | <0.001 | -0.203 | <0.001 | 0.106 | <0.001 |
|  |  | Binary | -1.899 | <0.001 | 0.583 | <0.001 | -0.603 | 0.001 |
|  | log(hover time) | Trunc. | -0.919 | <0.001 | -0.182 | <0.001 | 0.065 | 0.004 |
|  |  | Binary | 0.493 | 0.032 | 0.297 | <0.001 | -0.422 | <0.001 |
|  | hovers | - | -0.644 | <0.001 | -0.141 | <0.001 | 0.093 | 0.013 |
| Distance/Speed | | | | | | | | |
|  | total distance | - | -0.857 | <0.001 | -0.160 | <0.001 | -0.028 | 0.494 |
|  | log(max(vel.)) | - | -0.013 | 0.580 | 0.007 | 0.152 | -0.021 | 0.030 |
|  | log(max(vel.), time) | - | -0.764 | <0.001 | -0.167 | <0.001 | 0.078 | 0.007 |
|  | log(max(acc.)) | - | -0.001 | 0.981 | -0.006 | 0.385 | -0.007 | 0.606 |
|  | log(max(acc.), time) | - | -0.544 | <0.001 | -0.157 | <0.001 | 0.104 | <0.001 |
|  | log(min(acc.)) | - | -0.013 | 0.749 | 0.010 | 0.176 | -0.023 | 0.073 |
|  | log(min(acc.), time) | - | -0.465 | <0.001 | -0.136 | <0.001 | 0.078 | 0.001 |
| Entropy | | | | | | | | |
|  | log(entropy) | - | 0.025 | 0.677 | 0.029 | 0.014 | -0.044 | 0.047 |

SI Table 3. Coefficients and p-values for the three variables of interest relevant for the decision period but with Gain/Loss as predictor instead of Context. Significant values (here, p < 0.005) are indicated with a shaded background, red (lighter shade) when the coefficient is positive and blue (darker shade) when it is negative. Results from hurdle models are presented as two separate model parts, the binary part and the truncated part.

References

Dancey, C. P., & Reidy, J. (2007). Statistics without maths for psychology. Pearson education.

Hehman, E., Stolier, R. M. and Freeman, J.B. (2015). Advanced mouse-tracking analytic techniques for enhancing psychological science. *Group Processes & Intergroup Relations*, 18(3), 384-401. DOI: 10.1177/1368430214538325
